# Tear Biomarkers and Alzheimer’s Disease

**DOI:** 10.3390/ijms241713429

**Published:** 2023-08-30

**Authors:** Snježana Kaštelan, Marijana Braš, Neda Pjevač, Ivana Bakija, Zora Tomić, Nada Pjevač Keleminić, Antonela Gverović Antunica

**Affiliations:** 1 Department of Ophthalmology, Clinical Hospital Dubrava, School of Medicine, University of Zagreb, 10000 Zagreb, Croatia; 2 Centre for Palliative Medicine, Medical Ethics and Communication Skills, School of Medicine, University of Zagreb, 10000 Zagreb, Croatia; 3 Department of Medical Statistics, Epidemiology and Medical Informatics, School of Medicine, University of Zagreb, 10000 Zagreb, Croatia; 4 Department of Integrative Psychiatry, Psychiatry Hospital “Sveti Ivan”, 10090 Zagreb, Croatia; 5 Health Centre of the Croatian Department of Internal Affairs, 10000 Zagreb, Croatia; 6 Department of Family Medicine, Health Centre Zagreb-Centar, School of Medicine, University of Zagreb, 10000 Zagreb, Croatia; 7 Department of Ophthalmology, General Hospital Dubrovnik, University of Dubrovnik, 20000 Dubrovnik, Croatia

**Keywords:** tears, biomarkers, Alzheimer’s disease, dementia, neurocognitive disorder, early diagnosis, prognosis

## Abstract

Alzheimer’s disease (AD) is an age-related progressive neurodegenerative brain disorder that represents the most common type of dementia. It poses a significant diagnostic challenge that requires timely recognition and treatment. Currently, there is no effective therapy for AD; however, certain medications may slow down its progression. The discovery of AD biomarkers, namely, magnetic resonance imaging, positron emission tomography and cerebrospinal fluid molecules (amyloid-β and tau) has advanced our understanding of this disease and has been crucial for identifying early neuropathologic changes prior to clinical changes and cognitive decline. The close interrelationship between the eye and the brain suggests that tears could be an interesting source of biomarkers for AD; however, studies in this area are limited. The identification of biomarkers in tears will enable the development of cost-effective, non-invasive methods of screening, diagnosis and disease monitoring. In order to use tears as a standard method for early and non-invasive diagnosis of AD, future studies need to be conducted on a larger scale.

## 1. Introduction

Alzheimer’s disease (AD) is an age-related progressive neurodegenerative brain disorder. It results from neuron loss in the brain, primarily in the cortex leading to progressive behavioural, cognitive and motor damage [1]. AD represents the most common form of dementia, causing a high level of health impairment and mortality worldwide with limited treatment options available. The disease poses significant diagnostic challenges, requiring timely recognition and treatment, and has become a leading public health problem [1,2,3,4,5].

At the molecular level, the underlying pathophysiological mechanisms of AD include the extracellular deposition of amyloid-β (Aβ) peptides, known as amyloid plaques, as well as the intracellular development of hyperphosphorylated tau ((Tubulin-Associated Unit) protein aggregates, known as neurofibrillary tangles (NFTs). These processes subsequently trigger oxidative stress, chronic neuroinflammation, neuronal dysfunction and neurodegeneration [1,6]. When tau becomes hyperphosphorylated, its ability to bind to microtubules is reduced, resulting in abnormal aggregation into filaments. This, in turn, leads to microtubule collapse and compromised axonal transport [7]. Physiologically, there is a dynamic equilibrium between phosphorylation and dephosphorylation of tau protein within the cell body [8]. Excessive hyperphosphorylation of tau leads to the formation of insoluble NFTs, resulting in synaptic dysfunctions and neural cell damage [6]. In normal brain tissue, beta-amyloid is broken down and eliminated, whereas, in AD, the removal process is incomplete, leading to the formation of plaques that are characteristic of this condition. Other neuropathological features of AD include synaptic and neuronal loss, neuroinflammation with reactive gliosis, neuronal iron accumulation and the presence of cytoplasmic granulovacuolar degeneration bodies [9,10,11,12,13]. As a consequence of these processes, there is progressive atrophy of brain structures, including frontal, temporal and parietal lobes, entorhinal cortex, amygdala and hippocampus. Ultimately, these molecular and histopathological changes negatively affect cortical cognitive functions such as memory, motor skills and language abilities, potentially contributing to the development and exacerbation of depression or anxiety states [14]. 

AD is defined as a homogeneous central nervous system (CNS) disorder; however, considering its association with numerous physical and systemic changes affecting both the CNS and periphery, it can also be considered a multifactorial systemic disease [15]. The diagnosis of AD has been enhanced by the advancement of non-invasive neuroimaging techniques that allow the visualization of structures in vivo. Detection of the early stages relies on magnetic resonance imaging (MRI), functional MRI (fMRI), computed tomography (CT) scanning techniques, positron emission tomography (PET), amyloid imaging and cerebrospinal fluid (CSF) biomarkers [16,17,18,19,20,21]. Despite substantial progress in the understanding of AD, by the time clinical symptoms develop, therapeutic intervention is often untimely [1,21]. In the absence of diagnostic procedures for timely determination of AD onset, treatment options deem ineffective as neuronal damage at that stage becomes irreparable [22]. To date, while disease-modifying therapy remains unavailable, certain medications may aid in slowing or alleviating some symptoms. This underscores the importance of early symptom recognition and the timely initiation of treatment [1,21,23,24,25]. Currently, two treatments have been approved by the US Food and Drug Administration (FDA): Aducanumab, approved in June 2021, and Lecanemab, recently approved in June 2023. Both medications are monoclonal antibodies that target aggregated forms of Aβ plaques and remove these deposits from the brains of patients in the early stage. Other medications, including cholinesterase inhibitors and glutamate regulators, can help alleviate symptoms such as memory loss and confusion [1,14,24,26,27].

## 2. Alzheimer’s Disease and the Visual System

The concept of AD as a systemic multifactorial disease is further supported by both the structural and functional changes of the visual system observed in patients with AD. Among these patients, alterations in visual acuity and visual field, colour vision deficiency, movement perception, reduced contrast sensitivity and impaired ocular fixation is often present. Additionally, compromised vision–hand coordination and difficulties in visual analysis and synthesis as well as identifying objects is frequently observed [1,4]. As the retina is an extension of the CNS, it presents classical morphological hallmarks of AD. Structural alterations within the retina associated with AD include loss of retinal ganglion cells with subsequent thinning of the ganglion cell layer, a reduction in axon numbers leading to optic nerve atrophy and changes in retinal blood vessels. These changes have been verified through techniques like optical coherent tomography (OCT) and OCT angiography [4,14]. At the molecular level, the presence of Aβ plaques and tau deposits has been identified in the retina and lens. Additionally, certain research suggests that the occurrence of protein deposits in the eyes of AD patients is associated with aggregates in the brain [4,6,14]. Further, patients with AD may exhibit reduced corneal sensitivity and corneal nerve disfunction along with abnormal tear function due to their irregular distribution on the ocular surface and insufficient tear drainage. These disturbances could be associated with a decrease in the acetylcholine (ACh) levels, which is also a characteristic feature of AD [4]. Finally, several AD-related alterations have been found in ocular fluids, namely, aqueous and vitreous humour, particularly in tears [14,28]. 

## 3. Biomarkers for Alzheimer’s Disease

Typical biomarkers for AD include Aβ peptides and various forms of tau proteins. The metabolism of amyloid precursor protein (APP) generates several amyloidogenic peptides with the most prevalent ones being Aβ40 and Aβ42. The solubility of amyloidogenic peptides is low and, under physiological conditions in blood or CFS, their concentrations typically range from 4–400 pg/mL. The concentration of Aβ40 is usually higher than that of Aβ42 which is more amyloidogenic, exhibits greater cytotoxicity, forms fibrils more rapidly, constitutes a major component of senile plaques in AD and is more directly associated with AD dementia. However, despite these facts, numerous studies indicate that the Aβ42/Aβ40 ratio holds greater significance for the pathogenesis of AD than their absolute concentrations [4,17]. Tauopathy is an additional hallmark of AD, even though it can be present in several CNS diseases. Tau hyperphosphorylation represents a significant molecular anomaly in AD. The total tau protein concentration (T-tau) in the blood or CSF is a marker of neurodegeneration, while the phosphorylated form at Thr181 (P-tau181) is a typical indicator of AD in CSF [4,29].

Genetic markers for early-onset AD encompass mutations in the APP, presenilin 1 and 2 (PS1 and PS2) and tau genes. Among these genes most strongly linked to the late-onset form, the apolipoprotein E (ApoE) allele emerges as the most promising candidate [30,31]. However, genetic risk biomarkers are rarely used given that they lack relevant diagnostic or prognostic benefit. Neuroimaging techniques are both expensive and not readily available and therefore, not commonly used except to confirm a definite diagnosis. Consequently, there is a need for finding biomarkers that are accessible in body fluids [4,29].

CSF collection is an invasive procedure that carries risks for the patient, demanding the expertise of well-trained medical professionals. However, it could provide valuable information regarding the biochemical changes taking place in the brain during the preclinical stages of AD [32]. This has directed research towards finding identifying biomarkers that involve less invasive procedures [33]. In this regard, blood biomarkers could be utilized to identify individuals with risk of AD [34]. Even though blood is easily accessible, the activity of the blood–brain barrier must be taken into account in the presence of neurodegenerative conditions. The number of biomarkers derived from the CNS and their level in the blood are quite low, which can cause interference during analysis [29,33]. Due to the capability of molecules to migrate from the blood to saliva through mechanisms such as passive diffusion, active transport or microfiltration, saliva has potential to be a promising source of AD-related biomarker that could contribute to the early and precise diagnosis of AD. An additional advantage of utilizing saliva as a biomarker source is its accessibility, non-invasiveness and cost-effectiveness. However, the lack of standardized collection methods, pre-processing and storage protocol remains a challenge. The most significant potential AD biomarkers found in saliva include Aβ peptides, T-tau and P-tau, acetylcholinesterase, lactoferrin and trehalose, each associated with distinct AD-related pathophysiological mechanisms. Further research is necessary to validate the reliability and accuracy of these biomarkers in diagnosing AD [6,33,35]. The close relationship between the brain and eye suggests that tears could potentially serve as a source of AD biomarkers since they are easily accessible, having a basic and constant composition and allowing non-invasive collection procedures. Furthermore, the identification of early AD biomarkers in tears could be particularly valuable for screening in the general population [2,4,14,36,37]. Table 1 presents the advantages and limitations of individual biomarkers for AD. 

## 4. Tears as a Source of Biomarkers

Human tears are produced by the lacrimal glands, through blood plasma filtration, and comprise water, various biomolecules, proteins, tear lipid, mucin, nucleotides, vitamins, electrolytes and other components [38]. In healthy individuals, human tears contain 1351 proteins and total concentration that ranges from 6 to 11 mg/mL, with the most abundant being lysozyme with 1 mg/mL [39]. Other dominant proteins in tears include lactoferrin, lipocalin, secretory immunoglobulin A, superoxide dismutase, cystatins and a1-protease inhibitor, which, together with lysozyme, account for more than 90% of all tear proteins [40]. Moreover, potential biomarkers in tears encompass growth and neurotrophic factors, cytokines, cell adhesion molecules, immunoglobulins, sexual hormones, matrix metalloproteinases, proteases and protease inhibitors, calcium-binding proteins, glycoproteins and, particularly interesting, circulating microRNAs [38,39,40,41]. The tear fluid present on the eye’s surface forms a barrier that is part of the innate immune system. Antibacterial and immunomodulatory proteins (AMP) such as lipocalin-1, lactotransferrin and lysozyme-C are involved in immune and inflammatory processes, inhibit bacterial growth and provide defence against pathogens [42]. Tear composition undergoes constant alterations in response to various microbial and mechanical stimuli to ensure eye protection. In addition to local stimuli, systemic changes including AD can also affect the production and secretion of AMP and consequently their levels in tears resulting in changes of tears composition [39,42].

Since tears are a non-invasive biofluid, they are easy to collect and can be stored for extended periods they can be a valuable source of information relevant to various disorders. Numerous ocular (diabetic retinopathy, glaucoma, keratoconus, dry eye disease) and systemic disorders (thyroid disease, systemic sclerosis, diabetes mellitus, cystic fibrosis, cancer and neurological disorders: multiple sclerosis, Parkinson’s disease, AD, migraines) can be assessed and identified through biomarkers present in tears [39,41,43,44,45]. A particular challenge in utilising tears as a source of biomarker lies in the limited sample volume, concerns related to reproducibility and the potential for comparing different tear collection methods as well as the need for standardizing analytical techniques. Furthermore, during the process of tear collection, it is crucial to take into account the potential impact of circadian rhythms and environmental factors that might influence the levels of the biomarker being investigated. These factors can impact the accurate measurement and interpretation of biomarker levels, emphasizing the importance of considering these variables during the collection and analysis of tear samples. To date, there is limited available data concerning diurnal variations and fluctuations in the concentrations of different molecules in tears, as well as tear volume [46,47,48,49,50,51]. To ensure a valid comparison of tear cytokine levels between healthy and diseased individuals, a comprehensive understanding of physiological variations—both intra-day changes and inter-day variations are essential. The idea that levels of different molecules in tears can serve as potential biomarkers is based on the assumption that these levels do not undergo significant changes throughout the day or over an extended period. Studies demonstrating insignificant day-to-day variation further support their potential utility as biomarkers. In this regard, Benito et al. [51] have demonstrated that cytokines and chemokines in tears of healthy subjects exhibit reproducible measurements over time. Importantly, these tear cytokine and chemokine levels show no significant inter-day variability, rendering them suitable candidates as biomarkers. However, if circadian variations are confirmed, it is necessary to coordinate the sampling time. Additionally, it is established that various environmental factors, including temperature, humidity, air pollution, dust, wind and central heating or air conditioning, as well as digital device use, contact lens usage and the application of topical and systemic medications, can have a substantial impact on tear production, volume and composition. To ensure the accuracy of biomarker analysis, it is important to consider and address all of these factors [44,52,53,54,55,56]. Establishing a controlled environment during tear collection, where these variables remain constant, becomes crucial. This strategic approach aims to mitigate the potential influence of external factors such as circadian variations and environmental influences on research outcomes, thereby enhancing the reliability and accuracy of the results. Advancements in proteomic, lipidomic and glycomic techniques have enabled a more accurate analysis of tear components and a better comprehension of their association with various ocular and systemic diseases and disorders including AD [39,41,43,44,55]. The increasing prevalence of AD has emphasized the need for developing novel screening and early-diagnostic procedures with low cost and minimally invasive methods. Given this, analysis of tears has become a promising technique [39].

### Collection and Analysis of Tears

Due to recent advancement in collection and tear analysis techniques, numerous studies have proposed tears as indicators of both normal biological processes and pathogenic conditions. 

Tears may be collected using several methods such as Schirmer strips, micro-sponges, microcapillary tubes and micropipettes [4,44] (Table 2). Despite the limited volume of tear samples, proteomic and lipidomic advancements have facilitated more accurate analyses of tear elements, enhancing our comprehension of their roles in various diseases [45]. Analytical methods used include electrophoresis, spectrophotometric techniques, enzyme immunoassays (ELISA), microarrays and bead-based tests [56,57,58,59]. Additionally, there have been investigations into the use of biosensors and contact lenses for assessment purposes [4,60,61].

Analysing biomarkers in systemic circulation is a routine practice, yet applying the same process with ocular samples poses a challenge. Similar to other recently developed techniques, the analysis of tear samples lacks a standardized approach. Given the limited volume of the ocular sample and the complexity of the sampling process, it is important to establish standardized guidelines for methods of collection, storage, processing and analysis. Such guidelines would facilitate cross-study comparisons and strengthen the reliability of collected data. Inconsistencies in outcomes between studies could arise from differences in sample collection, storage methods and analytical processes. When collecting tears, it is important to avoid the activation of corneal nerves and reflex tearing, as these factors could potentially alter the composition of tear fluid. External factors, such as the application of topical anaesthesia, environmental conditions, timing and duration of sampling, daily fluctuations in tear volume, osmolarity and composition, the usage of artificial tears and the wearing of contact lenses can influence tear composition and potentially affect the interpretation of results. Moreover, collection techniques, sample storage, the complexity of the dilution process and subsequent reduction in analytical sensitivity of the method, along with the sample centrifugation process, analyte stability, validation, calibration procedures and the lack of reference intervals, can pose additional challenges that may impact the interpretation and reproducibility of the analytical results [44,49,52,62,63].

Managing patients with dry eye can pose a particular challenge. In cases of hypolacrimation, the only suitable sampling method involves collecting tear samples using a micropipette after flushing the eye surface with sterile distilled water. Alternatively, tear volume in the eye can be increased by obstructing tear drainage through the placement of punctal plugs [40,62,64]. Recently, neurostimulation of the lacrimal functional unit (LFU) has been employed to enhance tear production. Neurostimulation is a unique approach, aimed at enhancing the production of all basal tear components by stimulating the nerves responsible for their production. The neuroanatomy of the LFU provides several potential access points to stimulate tear production through two arms of the sensory trigeminal nerves. From a clinical perspective, neurostimulation has improved the signs and symptoms of dry eye by increasing basal tear production and tear volume. Several intranasal and extranasal devices for neurostimulation are presently under development and in use. Additionally, increasing endogenous tear production is achievable through pharmacological neuroactivation of the nasolacrimal reflex using intranasal spray varenicline, a highly selective nicotinic acetylcholine receptor agonist. The employment of neurostimulation through electrical, mechanical or chemical methods is a novel concept aimed at enhancing tear production [65,66].

## 5. Tear Biomarkers in Alzheimer’s Disease

### 5.1. Current Research

Based on the available data, research investigating tears as potential biomarkers for AD are limited [61,67,68,69,70,71,72] (Table 3). Total tear proteomic concentration levels, including changes in tear function and flow rate, have been described indicating autonomic nervous system dysfunction in individuals with AD [61,67].

Kallo et al. (2021) [67] conducted a study involving tear samples collected from both AD patients and healthy controls. They found a significantly increased flow rate of 12 ± 2 μg/min in AD patients compared to controls (6 ± 2 μg/min) as well as increased protein concentrations in tears of AD patients (8.8 ± 2.9 μg/μL) in comparison to healthy controls (4.4 ± 1.4 μg/μL). In their study, in order to measure tear proteins, they used the standard method of quantitative proteomics as well as electrophoresis and liquid chromatography-mass spectroscopy/mass spectroscopy (LC–MS/MS). They also found substantially reduced levels of lysozyme-C, lipocalin-1 and lacritin along with elevated dermcidin in the tears of AD patients. The combination of these factors has shown to be a prospective AD biomarker, showing sensitivity and specificity of 81% and 77%, respectively, for predicting the disease. Since lysozyme-C, lipocalin-1 and lacritin are primarily secreted by the lacrimal glands, they concluded that, in addition to neurodegenerative processes, dysfunction of the lacrimal gland might also be present in AD. Furthermore, the research also suggested that tear flow rate was significantly faster and total protein levels were more elevated in the tears of patients with AD compared to the controls.

In a study conducted by Del Prete et al. [69] a high level of Aβ42 protein was found in the tears of two healthy subjects with a family history of AD using immunocytochemistry assay. The presence of retinal plaques was directly correlated with the existence of Aβ42 in tears, whereas this association was absent in the tears of the healthy participant without family history. Considering that observed patients were phenotypically healthy, the identification of Aβ42 in tears could potentially be used for early diagnosis of AD as well as screening purposes.

The presence of Aβ40 and Aβ42 peptides in the tears of healthy individuals aged 20 to 79 was shown by Wang et al. [71]. They developed an innovative, inexpensive, disposable and user-friendly electrochemical immunosensor capable of detecting Aβ in tear specimens. They found that the amount of both peptides could be up to 10 times higher in the tear fluid (10 pg/mL level) than in blood (1 pg/mL level) and that Aβ concentrations could be age related.

The potential value of tau and Aβ proteins in tears as biomarkers of AD intensity was highlighted by investigations of tear amyloid and tau levels in correlation with neurodegeneration and AD severity conducted by Gijs et al. [70]. In their research, by employing multiple immunoassay platforms (triplet test for Aβ-38, -40 and -42 and duplex test for T-tau and P-Tau), they successfully identified five distinct molecules in tear samples. The detectability of amyloid peptides in tears was notably high only for the Aβ40 type in more than 94% of tear fluid samples. Aβ38 and Aβ42 were found in fewer than 23% of all samples, while Aβ42 was detected mainly in the healthy group. The measured levels of amyloid peptides were higher in the three test groups, with the median ranging from 17 to 1680 pg/mL, as compared to the healthy controls which showed medians of 4 to 60 pg/mL, although no significant differences were observed. Due to the low detectability of Aβ42, the Aβ42/Aβ40 ratio could not be estimated. Among patients showing neurodegeneration, classified according to the A/T/N system, the tear T-tau was found in 94% of samples. These levels were higher than in those without neurodegeneration and concentrations in tears were 10 times higher than those measured in CSF. Additionally, levels of P-tau were not measurable in the tear samples of the healthy controls [70]. This study shows a strong correlation between tau proteins in tears and disease severity, as well as neurodegeneration.

Another study exploring the potential diagnostic role of tau and Aβ proteins in tears was conducted by Gharbiya et al. [72]. They analysed the concentrations of β-amyloid peptide Aβ1-42, C-terminal fragment of amyloid precursor protein (APP-CTF) and P-tau in tears of individuals with MCI, mild to moderate AD and healthy subjects. Their analysis revealed that tear Aβ1-42 levels could identify both MCI and AD patients with a specificity of 93% and a sensitivity of 81%. No significant differences were noted in the relative abundance of APP-CTF and P-tau in tears. According to their results, evaluating the levels of Aβ1-42 in tears could serve as a minimally invasive method for early detection and diagnosis of AD. The presence of low levels of Aβ1-42 in tears may represent a specific, sensitive, non-invasive and inexpensive biomarker for early diagnosis of AD [72]. The significantly low levels of Aβ1-42 in tears found in this investigation are consistent with the level of this biomarker found in CSF. The reduced levels of Aβ1-42 in CSF are thought to be the result of peptide sequestration in the brain; similarly, the low level in tears might be attributed to sequestration within the lacrimal gland [73]. Previous research on the connection between CSF and tears in multiple sclerosis demonstrated the presence of oligoclonal bands in both samples, implying shared functions between the lacrimal glands and CNS lymphoid follicles [74,75]. Analogous mechanisms could underlie the reduced levels of Aβ1-42 in both CSF and tears. Beta-amyloid fragments have been detected in lacrimal glands, particularly in acinar cells. Consequently, the low levels of beta-amyloid in tears from AD patients might be due to the increased storage of peptide fragments in gland cells such as neutrophil granulocytes [76,77].

Kenny et al. [68] conducted an investigation into the microRNA profile in the tears of patients with cognitive impairment and revealing that the total microRNA-200b-5p tear concentration was highly up-regulated in the AD group in relation to the controls. The elongation initiation factor 4E (eIF4E), a polypeptide implicated in various cellular processes including protein synthesis, mRNA stability and RNA nuclear export, was present only in the tears of AD patients. The analysis of proteins in tears was carried out using liquid chromatography–mass spectrometry (LC–MS), while microRNA was assessed using a genome-wide high-throughput polymerase chain reaction-based platform. To fully comprehend the relationship between AD and microRNA-200b-5p and eIF4E, further investigations are required.

The findings from these studies strongly suggest that tears could potentially serve as a valuable collection base for biomarkers related to AD. Detecting such biomarkers within tears has the potential to facilitate the development of a non-invasive and cost-effective test for the early recognition of AD.

### 5.2. MicroRNAs as Potential Biomarkers for AD

MicroRNAs (miRNAs) are endogenous, short non-coding RNAs that play important role as regulators of overall gene expression, serving as regulatory molecules in various biological pathways. Extracellular miRNAs have been detected in various biofluids, making them potential candidates for diagnostic purposes and biomarkers. Circulating miRNAs have shown great stability and resistance to degradation even during extended storage and multiple freeze–thaw cycles, which is a good foundation for their potential clinical use. Moreover, due to the inherent characteristics and structure of miRNAs, as well as the feasibility of direct detection methods, they represent a promising source of biomarkers [78]. To date, in tear fluid, approximately 300 different miRNAs have been isolated. Among these, some are newly discovered and their regulatory roles remain relatively unexplored [68,78,79].

Extracellular circulating miRNAs have been investigated as potential biomarkers for AD diagnosis in various studies given their recognized significance in neuronal function and survival [68,78,80,81,82]. Research has demonstrated that miR-200b/c might play a role in decreasing Aβ secretion and mitigating Aβ-induced cognitive impairment in primary neurons of AD mouse models. Altered miRNA profiles potentially signify a defensive response against the pathogenesis of neurodegenerative disorders, such as AD [80]. In another study, negative correlations were observed between miR-384 and Aβ42 levels in the serum and CSF of AD patients, suggesting that miR-384 may play a role in the development of AD [64]. Further, Wijesinghe et al. [82] investigated 10 miRNA candidates as potential biomarkers of AD in neocortex–hippocampus, eye tissue and tear fluid samples using a murine model. According to their results, miRNAs associated with Aβ production (-101a, -15a and -342) and proinflammation (-125b, -146a and -34a) showed significant up-regulations in the tear fluids with disease progression, as tracked by cortical Aβ load and reactive astrogliosis. The findings from Li et al. [83] suggest that miR-128 plays a role in suppressing the development of AD and may serve as a promising therapeutic target. Their research also proposed a mechanism to explain the dysregulation of miR-128 in AD. According to this mechanism, Aβ contributes to the downregulation of miR-128 expression by inhibiting C/EBPα [83]. Research conducted on a mouse model of AD has demonstrated that the dysregulation of miR-200c expression contributes to the pathogenesis of AD, leading to cognitive impairment through the promotion of tau phosphorylation. Furthermore, atypical expression of miR-200c has also been confirmed in the blood of individuals with AD [84]. Elevated levels of miRNA-146a have been identified in the neocortex and superior temporal lobe hippocampus of individuals with AD, as well as in stressed primary co-cultures of human neuronal–glial (HNG) cells and transgenic AD animal models. These findings emphasize the potential significance of this NF-κB-regulated, brain-enriched miRNA species in neurodegenerative diseases. Additionally, it has been confirmed that the expression of miRNA-146a correlates with the density of senile plaques and synaptic pathology in both Tg2576 and 5xFAD transgenic mouse models [85]. He et al. [86] conducted a study to investigate the abundance and complexity of miRNAs in AD brain tissues, comparing them with age-matched controls. They observed a consistent up-regulation of several brain-enriched miRNAs, namely, miRNA-9, miRNA-34a, miRNA-125b, miRNA-146a and miRNA-155, both in short post-mortem AD brain samples and stressed primary HNG cells under the transcriptional control of the pro-inflammatory transcription factor NF-kB. Among the inducible miRNAs within this subfamily, miRNA-125b stands out as one of the most abundant and significantly induced species of miRNA in human brain cells and tissues. This finding could be particularly significant since pro-inflammatory miRNAs, such as miRNA-125b, appear to carry unique pathogenetic signalling information that can drive AD-type pathology in adjacent brain cell types [86]. With recent advances in detection methods, studies investigating miRNAs becoming increasingly significant due to biological relevance and extracellular stability of miRNA, as well as the possibility of their detection in tears, which provide additional advantages in terms of accessibility [78,80,81,82,83,84,85,86].

### 5.3. Lactoferrin as a Potential Biomarker for AD

Lactoferrin (LF) is one of the main functional proteins that plays a significant role in maintaining human health due to its antioxidant, antibacterial, antiviral, anti-inflammatory and neuroprotective activities. In terms of its neuroprotective effects, it interacts with the brain and has been shown to play a role in the progression of Alzheimer’s and Parkinson’s disease [87,88]. It has been shown to bind Aβ and is detected in high concentrations in neurons and glial cells, Aβ senile plaques and NFTs within the brain of patients with AD [89]. LF can be found in serum, CSF, milk, tears, faeces and other secretions within the human body. It has been identified as a biomarker indicative of several diseases, such as AD and dry eye disease [88,89]. It plays a pivotal role in various physiological functions, including iron binding and transport, neuroprotective effects, regulation of immune responses, anti-inflammatory properties and antioxidant and anticarcinogenic activities [90]. The antimicrobial properties of LF are attributed to its highly positively charged N-terminal region [91], which enables it to act as a first line of defence against bacteria, viruses, fungi, free radicals, protozoa and yeasts [89,91,92]. LF is present in neurons and glia cells and has been identified in senile plaques, NFTs and microglia within the brain of individuals with AD [87,88,92].

According to certain theories, bacterial and viral infections could potentially contribute to the development of AD by compromising the function of the innate immune system. Several preclinical and clinical studies have demonstrated the involvement of LF in the pathogenesis of AD. Wang et al. [93] conducted immunohistochemical investigations to determine the localization of LF in the brains of APP-transgenic mice, which serve as a model for AD. No LF immunoreactivity was detected in the brains of wild-type mice. However, LF deposition was observed in the brains of transgenic AD mice. Through double-immunofluorescence staining with antibodies targeting the Aβ peptide and LF, the LF depositions were found to be localized to amyloid deposits (senile plaques) as well as regions displaying amyloid angiopathy. Senile plaque formation was observed to occur before the deposition of LF in AD. Among transgenic mice under 18 months of age, a majority of senile plaques lacked LF presence. At the age of 18 months, weak LF deposits began to emerge in these mice. Subsequently, both the intensity and quantity of LF-positive depositions in the transgenic mice exhibited an increase with age. The observed up-regulation of LF in the brains of both AD patients and the transgenic mouse AD model presents compelling evidence for the significant role of LF in brain tissues affected by AD [94]. Several animal models were used to investigate the impact of LF administration on cognitive function. The study assessing the effects of LF on the cognitive ability of 16-month-old C57/BL6J mice demonstrated the potential of LF to protect and enhance cognitive function in aged animals. These findings offer a unique pharmacological approach for addressing neurodegenerative disorders associated with aging [94]. The study conducted by Guo et al. demonstrated that human LF (hLF) treatment can improve cognitive deficits and reduce Aβ aggregation in APP/PS1 mice after 90 days of administration. Further investigations revealed that hLF can induce the activation of a-disintegrin and metalloprotease 10 (ADAM10) and facilitate non-amyloidogenic α-secretase processing of APP by engaging the ERK-CREB and hypoxia-inducible factor 1α (HIF-1α) signalling pathways in both APP/PS1 mice and N2aSW cells. Additionally, hLF treatment reduced oxidative stress and neuroinflammation in the brain of APP/PS1 mice. These findings provide a mechanistic understanding of the potential therapeutic effectiveness of hLF in treating AD. The obtained results offer valuable insights into the potential therapeutic application of hLF for the treatment of AD [95]. In the study that investigated the effects of LF on memory impairment and AD pathogenesis in AβPP-Tg mice (J20 mice), researchers discovered that both LF and pepsin-hydrolyzed LF (LF-hyd) diets mitigated memory impairment and reduced brain Aβ40 and Aβ42 levels. This reduction was attributed to the inhibition of amyloidogenic processing of AβPP, resulting in a decrease in β-site amyloid protein precursor-cleaving enzyme 1 (BACE1) levels. Additionally, LF and LF-hyd treatments were found to elevate both ApoE secretion and ATP-binding cassette A1 (ABCA1) protein levels in the brains of J20 mice and primary astrocyte cultures. Moreover, LF and LF-hyd facilitated the extracellular degradation of Aβ in primary astrocyte cultures. These findings suggest that the decline in Aβ levels observed in the brains of mice fed with both LF and LF-hyd diets could be influenced by the increased ApoE secretion and elevated ABCA1 protein levels. This, in turn, could lead to enhanced Aβ degradation within the brains of J20 mice. Overall, these findings indicate the potential of LF and LF-hyd as therapeutic options for treating and preventing the development of AD [96]. Considering that LF is a significant defensive component in saliva due to its antimicrobial properties, several studies have explored the levels of LF in saliva and its potential correlation with AD [89]. Antequera et al. [97] investigated the levels of salivary LF in a mouse model of AD and observed a significant and early decrease in 6- and 12-month-old APP/PS1 mice. They proposed that this reduction could be attributed to impaired ACh release, resulting in decreased ACh binding to muscarinic M3 receptors. This sequence of events could subsequently lead to a diminished secretion of LF in the saliva of individuals with AD. Based on these findings, it is suggested that salivary LF may be a valuable biomarker for AD [97]. In their research Carro et al. [98] suggested that salivary LF could serve as a valuable diagnostic tool for AD. They observed a decreased concentration of LF in the saliva of AD patients compared to controls and the results were more accurate than those obtained by analysing established biomarkers such as total tau and Aβ42 in CSF. This study also showed that apparently healthy participants with low salivary LF levels would have a relatively high likelihood of developing AD in the future. Therefore, it appears that salivary LF levels could be used for the early identification of individuals at risk for developing MCI and AD with a sensitivity of 100% and a specificity of 98.6%. Ultimately, the authors provided evidence supporting the potential to predict the development of MCI and AD in healthy subjects based on salivary LF levels [97]. Another study conducted by González-Sánchez et al. [99] utilized salivary LF to diagnose prodromal AD and examined the correlation between salivary LF and cerebral Aβ. The result revealed that the salivary LF levels decreased only in patients with MCI and AD and these levels were correlated with the amyloid PET imaging profile, as opposed to other types of dementia [99]. Given that LF is a significant defensive element in tears and is recognized as a biomarker for various disorders, there is potential value in conducting investigations to explore LF as a possible tear biomarker for AD in the future.

## 6. Future Perspectives

**Figure 1:**
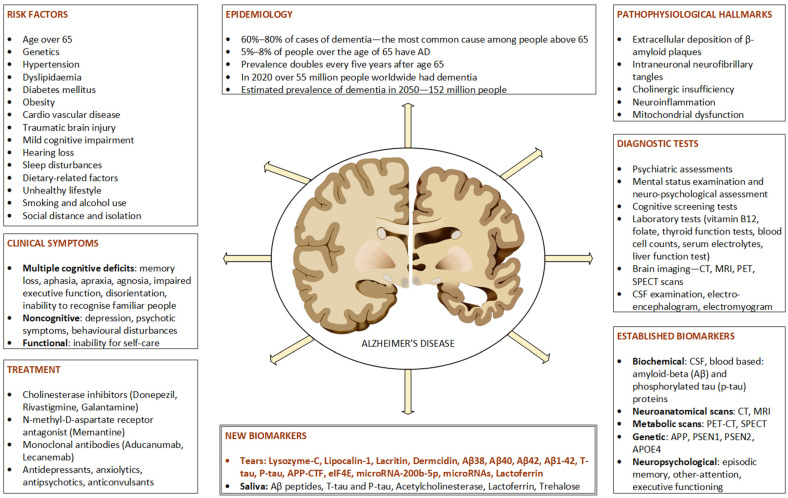
Clinical and diagnostic features of Alzheimer’s disease. AD: Alzheimer’s disease; CT: computerized tomography; MRI: magnetic resonance imaging; PET: positron emission tomography; SPECT: single-photon emission computed tomography; Aβ: amyloid-beta; CSF: cerebrospinal fluid; APP: gene for amyloid precursor protein; APOE4: apolipoprotein E4 allele; PSEN1, PSEN2: presenilin gene 1 and 2; CSF: cerebrospinal fluid.

There is a global demand for accurate and early diagnosis of AD. However, clinical diagnosis of MCI or early dementia is complex due to its subtle onset and gradual cognitive decline. The difficulties in timely identification and consistent tracking the disease progression, along with assessing therapeutic responses, underscore the need for a reliable and easily accessible biomarker to enhance clinical care and facilitate the advancement of disease-modifying treatments [100]. Figure 1 gives a detailed description of epidemiological, clinical and diagnostic features of AD.

The identification of AD biomarkers, such as PET imaging and CSF molecules (Aβ and tau), has significantly enhanced our comprehension of the disease and is essential for recognizing initial neuropathological alterations preceding clinical and cognitive decline. Nevertheless, these methods are costly and involve invasiveness, thereby posing challenges to widespread population screening and implementation [1,2,4]. Considering the challenges related to brain accessibility and the expense of diagnostic methods, there is a rising emphasis on investigating more readily accessible bodily fluids for AD diagnosis, such as saliva and, notably, tears. These bodily fluids have the potential to serve as sources of diagnostic and prognostic biomarkers for various neurological disorders [101]. Given that the eye is an extension of the CNS, investigating changes in ocular biology could lay the foundation for developing of a range of non-invasive, differential tests for AD diagnosis, as has been demonstrated in other diseases [2,102]. In patients with AD, specific ocular changes are evident including a reduction in the count of retinal ganglion cells, thinning of the nerve cell layer, a decrease in the number of axons and the accumulation of amyloid within the lens and retina. It is conceivable that these ocular changes induced by AD could potentially influence the function of the lacrimal gland, subsequently affecting tear production and the composition [101]. Tear biomarkers for AD are still in their early stages and are not without limitations. However, they offer the advantage of rapid, simple and repeatable tear sample collection without the necessity for specialized training, equipment or invasive procedures. Consequently, tear fluid analysis introduces novel possibilities for research and diagnostics in many fields of medicine. It is worth noting that distinct sampling methods carry specific advantages and limitations. Thus, comparing different approaches for collecting tear fluid and assessing their suitability could prove valuable when addressing specific research inquiries [39,43,103]. Current research suggests that potential tear biomarkers for AD include lipocalin-1, lysozyme-C, lacritin, eIF4E, Aβ38, Aβ40, Aβ42, Aβ1-42, T-tau and P-tau [67,68,69,70,71,72]. Additionally, miRNAs and lactoferrin emerge as promising biomarkers for AD, showing particular potential. Nonetheless, conducting comprehensive research is essential to validate these biomarkers and determine whether they are specific to AD or indicative of neurodegeneration in general. However, to obtain reliable results that enable meaningful conclusions to be drawn, it is very important to include an adequate number of AD patients in the planned research. The groups of included patients must be well defined based on clear and well-specified diagnostic criteria. One set of them is the NIA–AA Alzheimer’s criteria, which were developed by the National Institute on Aging and the Alzheimer’s Association. These criteria take into consideration clinical symptoms as well as the presence of biomarkers in CSF, PET and other advanced imaging techniques, all of which contribute to enhancing diagnostic certainty. The NIA–AA criteria have become increasingly used in research studies and clinical trials owing their ability to encompass the different stages of AD pathology and their emphasis on early diagnosis through biomarkers. It is important to note that the criteria for AD diagnosis and staging undergo changes as research progresses and our understanding of the disease grows [104,105]. In this regard, biomarkers in tears are becoming promising for improving the accuracy of diagnosis, monitoring disease progression and determining the success of treatment. Establishing commercial screening tests aimed at finding prompt diagnosis will enable extensive and detailed examinations in order to determine the precise diagnosis as well as implementation of timely treatment.

## 7. Conclusions

Tear biomarkers could provide the potential to offer a non-invasive approach for screening and monitoring of diseases, with significant implications for the development of new therapies, particularly during the preclinical stages of AD. Progress in tear sampling and analysis establishes a good foundation for future research in this rapidly evolving field, given that tears provide a wealth of information and are easily accessible. This underscores the importance of current advancements, challenges and forthcoming pathways in utilizing tears for early AD diagnosis. The various molecules discussed in this paper could serve as the foundation for clinical research; however, current data are insufficient to definitively establish their reliability as AD biomarkers. To establish tears as a standardized method for early and non-invasive AD diagnosis, future investigations must be conducted on a broader scale.

## Figures and Tables

**Table 1:** Advantages and limitations of biomarkers for Alzheimer’s disease.

Biomarker	Advantages	Limitations
**Genetic**	Insights into an individual’s genetic predisposition Insights into pathogenesis Early detection and risk assessment Development of personalized treatment strategies Clinical trial recruitment	Incomplete penetrance and gene expression Complex interactions with other factors Limited predictive accuracy Cost and accessibility Lack of treatment options Rarely used for routine clinical diagnosis of AD
**Brain imaging** **CT**	Provide detailed structural information Preferable to MRI for non-collaborative patients Relatively low cost Greater accessibility in developing countries	Not sensitive to early changes Limited quantitative measurements Lack of functional information Radiation exposure Inferior soft tissue detail Not considered as a standard biomarker for early diagnosis of AD
**Brain imaging** **MRI**	Insight into the microstructural, functional and molecular alterations occurring in the brain (DTI, MRS, fMRI) No radiation exposure Early diagnosis Quantification of brain atrophy Longitudinal monitoring of disease progression Distinguishing AD from other neurodegenerative disorders	Cost and availability Time consuming Patient cooperation Complexity of interpretation: expertise is required for the analysis of images Limited molecular information in comparison to PET scans Limited specificity and overlap with ageing Potentially missing early disease-related alterations Contrast side effects
**Brain imaging PET scan**	Early detection allowing for timely intervention and treatment planning Objective measurement of the extent and distribution of beta-amyloid and tau pathology in the brain Differentiation of AD from other forms of dementia Tracking disease progression Clinical trial recruitment	Cost and availability Exposition to ionizing radiation Time consuming Possibility of false positive and false negative results Ethical considerations in asymptomatic individuals Complexity of interpretation: expertise is required for the analysis of images Contrast side effects
**Cerebrospinal fluid**	Proximity to the brain—contains brain proteins High concentration of biomarkers Capacity to test numerous potential biomarkers Standardized methodology High accuracy in the diagnostic procedures Potential detection of AD in its early stages, even before significant clinical symptoms Longitudinal monitoring of disease progression Can be used as outcome measures to assess the efficacy of potential therapies	Invasive procedure Requires hospitalization Need for specialized expertise and equipment for collection and analysis Relatively high cost Risk of complications (infection, headache) A less acceptable procedure among the general population A risk of inducing harm, fear and anxiety in the patient Complex interpretation, as biomarker levels may vary due to age, gender and underlying health conditions
**Blood**	Minimally invasive and simple sampling Cost- and time-efficient Widespread use Reproducible and simple to measure Easy to implement in large populations Ability to test a large number of biomarkers The possibility of repeated sampling and measurements Initial diagnostic examination in a complex diagnostic procedure	Relatively low concentration of the potential biomarkers due to the presence of the blood–brain barrier Significant dilution of analytes caused by the volume ratio between the blood and the CSF Unreliability of findings—blood is a complex fluid—non-specific biomarkers may be expressed from sources other than the CNS Possible influence on the concentration levels of biomarkers due to liver or plasma proteolytic degradation, plasma protein or blood cell adhesion and kidney excretion The sensitivity and specificity of blood biomarkers for AD are still considerably low
**Saliva**	Easily accessible and non-invasive collection technique Repeatable collection—opportunity to monitor biomarker fluctuations Cost-effective and minimally stressful Without risk of infection Suitable for a wide range of individuals Convenient and reproducible sample collection Accessibility without regard to location or time limitations	Lack of standardisation in collection procedures (stimulated versus unstimulated samples), pre-processing and storage of samples Lack of validated studies Lack of results replicated in broader, multicentre and longitudinal investigations Difficulties in collecting saliva due to poor compliance of elderly patients, particularly with AD Limited sensitivity and specificity Saliva is a pooled sample from different salivary glands—potential influence on the sample composition Influence of ageing, oral health, circadian variations, environmental factors, psychogenic disorders, medication use, local and systemic pathology and treatment
**Tears**	Close association between eye and the brain Easily accessible and non-invasive collection technique Repeatable collection—opportunity to monitor biomarker fluctuations Cost-effective and minimally stressful Without risk of infection Suitable for a wide range of individuals Convenient and reproducible sample collection Accessibility without regard to location or time limitations	Lack of standardisation in collection procedures, pre-processing and storage of samples Lack of validated studies Lack of results that have been replicated in broader, multicentre and longitudinal investigations. Small volume sample size Tear production and drainage can influence the concentration of biomarkers The influence of circadian rhythms and environmental factors

AD: Alzheimer’s disease; MRI: magnetic resonance imaging; DTI: diffusion tensor imaging; MRS: MR spectroscopy; fMRI: functional MRI; CNS central nervous system.

**Table 2:** Characteristic of tear sampling methods.

Sampling Method	Description	Advantages	Disadvantages
Schirmer’s strip	Strips of sterile filter paper with an imprinted graduated scale Placed inside the lower eyelid with or without anaesthetics (basal or reflex tears)	Simple application Available in routine clinical practice	Unsuitable for dry eye Causes discomfort Demanding sampling processing Requires tear fluid extraction Retention of proteins
Microcapillary tube	Small diameter thin-walled tubes with capillary action Collect a volume of 10 µL within 10 minutes Gently placed to the lower eyelid	Comfortable procedure less frequent and shorter sensation of foreign body Convenient sampling collection method Easy post-collection procedure No elution necessary	Unsuitable for dry eye Risk of reflex tearing Requires experience Time-consuming process
Cellulose micro-sponge	Sponges with a high absorption rate Insertion—an inferior cul-de-sac of the eye, the eye surface of the inferior lower lid	Comfortable procedure (children) Time-saving Highly efficient Good reproducibility	Unsuitable for dry eye Demanding sampling processing Retention of proteins Inability to accurately determine the volume of tears
Micropipette	Tear samples collected using a micropipette after a flush of sterile distilled water (20 µL) over the eye surface	Suitable for dry eye Comfortable procedure Time-saving	Questionable reproducibility Diluted sample

**Table 3:** Clinical studies of tear biomarkers related to Alzheimer’s disease.

Author (Year)	Biomarker	AD Related Changes	Collection Method	Analytical Method	Results
Kallo et al. (2016) [67]	Lysozyme-C, Lipocalin-1 Lacritin Dermcidin	↓ ↓ ↓ ↑	Microcapillary tube	Electrophoresis LC–MS/MS analysis SRM-based targeted proteomics mass spectrometry	Significantly increased tear flow rates in AD patients. Significantly increased total protein concentration in tears of AD patients. Combination of lysozyme-C, lipocalin-1, lacritin and dermcidin could be a potential biomarker for AD, with 81% sensitivity and 77% specificity.
Kenny et. al. (2019) [68]	microRNAs microRNA-200b-5p eIF4E	↑ ↑ Present	Schirmer’s strips	Reverse phase-liquid chromatography RP-LC–MS/MS analysis Genome-wide high-throughput qPCR-based microRNA platform (*Open Array*)	Total microRNA abundance higher in AD patients. Elevated microRNA-200b-5p levels in tears of AD patients—potential biomarker. eIF4E existing only in AD patients—potential biomarker.
Del Prete et al. (2021) [69]	Aβ42	↑	Not reported	Immunocytochemistry assay	Aβ42 protein was found in the tear fluid of healthy subjects with a family history of AD. Relationship between the retinal plaques and the expression of Aβ42.
Gijs et al. (2021) [70]	Aβ38 Aβ40 Aβ42 T-tau P-tau	↑ ↑ ↑ ↑ ↑	Schirmer’s strips without topical anaesthesia	Multiplex immunoassay	Levels of Aβ40 and T-tau detectable in tear fluid of 94% of patients with cognitive impairment and correlated with cognitive decline. Tear T-tau levels elevated in patients with neurodegeneration. Tear T-tau concentrations were found to be ten times higher than measured in CSF.
Wang et al. (2021) [71]	Aβ40 Aβ42	NA	Schirmer’s strips	Electrochemical immunosensor	New biosensor testing. The concentration of Aβ peptide in tears of healthy people (10 pg/mL) was 10 times higher than in blood (1 pg/mL). Aβ concentration in healthy subjects was inversely proportional to age.
Gharbiya et al. (2023) [72]	Aβ1-42 APP-CTF P-tau	↓ NS NS	Micro-sponges	ELISA Western blot	Aβ1-42 levels in tears were lower in patients with mild cognitive impairment (*p* < 0.01) and with AD group (*p* < 0.001) compared to healthy controls. No differences were observed in the concentration of APP-CTF and P-tau in tears.

AD: Alzheimer’s disease; ↓: decrease; ↑: increase; LC–MS/MS: liquid chromatography–tandem mass spectrometry; SRM: selected reaction monitoring; RP-LC–MS/MS: reversed-phase liquid chromatography–tandem mass spectrometry; eIF4E: elongation initiation factor 4E; Aβ: Amyloid beta; T-tau: total tau; P-tau: phosphorylated tau; CSF: cerebrospinal fluid; APP-CTF: C-terminal fragment of amyloid precursor protein; NA: not applicable; NS: not significant; ELISA: Enzyme-Linked Immunosorbent Assay.
